# An Audit of Sport Nutrition Services Within Male and Female International Rugby Union: Implications for Research and Practice

**DOI:** 10.1002/ejsc.12260

**Published:** 2025-01-30

**Authors:** Lara Wilson, Ben Jones, Susan H. Backhouse, Andy Boyd, Nessan Costello

**Affiliations:** ^1^ Carnegie School of Sport Leeds Beckett University Leeds UK; ^2^ Scottish Rugby Union Murrayfield Stadium Edinburgh Scotland; ^3^ Division of Physiological Sciences Department of Human Biology Faculty of Health Sciences University of Cape Town Cape Town South Africa; ^4^ School of Behavioural and Health Sciences Australian Catholic University Brisbane Australia; ^5^ England Performance Unit Rugby Football League Manchester UK; ^6^ Rugby Department Premiership Rugby London UK

**Keywords:** athletes, sport science, team sport

## Abstract

To critically evaluate sport nutrition services available to male and female international rugby unions. Fifteen participants, representing 16 international rugby unions, including nine female and seven male teams (one participant worked with both a female and male union), responded to an online survey. Twelve of the unions recruited were ranked in the top 10 globally by World Rugby. Twelve unions employed accredited nutrition practitioners with significant experience (> 5 years: *n* = 5; > 10 years: *n* = 4) and advanced qualifications (master's degrees: *n* = 8; doctorates: *n* = 2). Three unions did not employ a qualified nutrition practitioner (female: *n* = 2; male: *n* = 1). Full‐time employment was more common among nutrition practitioners serving male (*n* = 4/5) versus female (*n* = 3/6) unions. Practitioners served male unions for more hours per week (42 ± 28) than female unions (24 ± 20). Practitioners were involved in sport science meetings (*n* = 14/15), anti‐doping education, menu design, strategy development (*n* = 13/15), body composition assessments, individual consultations (*n* = 12/15), focusing on fuelling, recovery and injury rehabilitation (*n* = 14/15). Participants were “*moderately confident*” (*n* = 8/15) in using behaviour change techniques. Most participants agreed on the lack of female‐specific nutrition guidance (*n* = 14/15), relying on guidance for male players due to limited evidence (*n* = 7/9). This study provides the first critical reflection of sport nutrition service delivery within international rugby. The findings highlight gender disparities for female players, with reduced applied support and a lack of female‐specific guidelines. Recommendations include enhancing practitioner training in behaviour change, hiring qualified nutritionists, deemphasising body composition assessment, and conducting more research to improve nutrition services, especially for women.

**Summary**

- Practitioners were experienced (11 ± 6 years), with all nutrition practitioners (*n* = 12) holding professional registration. However, three unions lacked accredited nutrition professionals, and nutrition service provision was devolved to other sports science and medicine (SSM) practitioners (e.g., S&C coaches and doctors). Employment of full‐time nutritionists was more common in male (*n* = 4/5) than female (*n* = 3/6) unions. Overall, nutritionists dedicated significantly more service hours (+23.1 h weekly) to male unions.
- Findings indicate a bias towards in‐season body composition assessment and management (male, *n* = 6/6 vs female, *n* = 5/8), potentially at the expense of broader nutritional requirements relating to athlete health and performance.
- The study highlights a deficiency in tailored nutrition guidance for female players (*n* = 14/15), with practitioners working with female players often relying on research primarily derived from male athletes (*n* = 7/9).
- Practitioners displayed varying confidence in employing behaviour change techniques (BCTs), with high confidence linked to positive relationships with players, observed nutritional changes and career experience. Commonly used BCTs included goal setting and problem solving. Several practitioners (*n* = 5/15) felt they required more training in behaviour change.

## 1 Introduction

Sports nutrition practitioners working within international rugby play a crucial role in safeguarding athlete health and performance by delivering high‐quality nutrition services (Close, Kasper, and Morton 2023; Chantler, Martin, and Sutton 2021; Costello, Chantler, and Hannon 2022). Although the value of nutrition in rugby is recognised, there is a shortage of detailed research on the actual services provided. Significant work has been done on establishing the dietary needs of athletes in various team sports (Mujika et al. 2011; Holway et al. 2013; Williams et al. 2015), including rugby (Hudson et al. 2019; Costello et al. 2019; Costello et al. 2018a; Smith et al. 2018; Morehen et al. 2016; O'Neill et al. 2022), yet the specific services available to rugby players at the international level remain unexplored. It is essential to conduct further research into the occupational conditions, roles and responsibilities and challenges of nutrition practitioners within this field. Such insights are vital for enhancing the support available to sports nutrition professionals and, consequently, the athletes they serve.

Female participation in rugby has experienced remarkable growth in recent years, accompanied by increased professionalism, infrastructure development, investment and global exposure (Rugby 2022). Despite this progress, differences exist between the male and female game (Fink 2015; Emmonds, Heyward, and Jones 2019), potentially extending to the provision of sport science services, including nutrition. Female athletes often face challenges such as limited funding, restricted training opportunities, and inadequate access to expert staff such as nutritionists, resulting in potential delegation of nutrition responsibilities to unaccredited professionals (Gilbert 2008; Jacob et al. 2019). Female athlete representation in sport science and medicine research remains limited. Although practical experience and anecdotal observations contribute to evolving knowledge (Jones et al. 2019), further research is now required to support improved nutrition service delivery in female rugby environments.

Therefore, the primary aim of this study was to evaluate the sport nutrition services available to international rugby players. A secondary aim was to explore any disparities in nutritional support provided to male versus female players.

## 2 Methods

### 2.1 Study Design, Participants and Recruitment

In a cross‐sectional research design, sports nutrition practitioners working with senior international male or female rugby unions were purposefully recruited to complete a closed survey. Data were collected between the 1st of December 2020 until the 2nd of February 2022, until at least 10 of the top 10 international rugby unions in the world had been recruited (minimum five female and five male unions; as of World Rugby Rankings, date accessed: 10/01/2022).

From a possible sample of 40 unions (top 20 world‐ranked male and female unions), the contact details of 29 practitioners, including 15 female and 14 male unions were obtained. Of the 29 practitioners, 20 practitioners agreed to participate, and were emailed the survey link. Of the 20 recruited practitioners, 15 completed the survey, and 5 practitioners did not. The eligibility criteria for participant recruitment included: (a) lead practitioner responsible for the nutrition service delivery at a senior international rugby union, and (b) should be aged 18 or over. Completion of the survey was voluntary, and no incentives were provided. Participants were informed of the benefits and risks of the investigation prior to signing an institutionally approved informed consent document to participate in the study.

In situations where an accredited sports nutritionist or dietician (i.e., holding a registration with the UK Sport and Exercise Nutrition Register or equivalent) was not present, then a relevant stakeholder (e.g., sport science and medicine staff) who met the eligibility criteria was recruited. Ethical approval for this study was obtained from Leeds Beckett University's Research Ethics Committee (approval ref: 84504).

The recruitment process followed a standardised approach. Initially, the lead researcher contacted the head of performance and/or medicine from international male and female rugby unions, who acted as the primary gatekeeper. Gatekeepers were contacted directly by email, which were either available online, known to the research team, or provided by other gatekeepers. In cases where the primary contact with gatekeepers was unsuccessful (*n* = 10), then other relevant club staff were approached, such as sports science and medical personnel, as secondary gatekeepers.

If no response was received from either the primary or secondary gatekeeper (*n* = 5), then the lead researcher contacted sport nutrition practitioners directly through email or other platforms (e.g., LinkedIn). Practitioners known to the research team (*n* = 3), were contacted directly via email. To maintain data integrity and prevent duplicate responses, only one practitioner was invited to participate from each male or female union.

Email communications detailed who the lead researcher was, the purpose of the research, the anticipated survey completion time and data protection and storage information. These details were further expanded upon within the participant information sheet attached to the email. Follow‐up correspondence was sent out via email 3 weeks after initial communication. Following agreement to participate, each participant was provided with the survey link. Further email prompts were sent 2 weeks after survey link circulation, to encourage questionnaire completion.

The first page of the survey displayed the participant information sheet. Informed consent was obtained online prior to starting the survey. To prevent duplicate submissions and maintain the integrity of the study results, internet protocol (IP) addresses were collected. If duplicate entries from the same IP address were submitted within a 48‐h period, the first entry was eliminated before analysis. There were no duplicate entries in this research. To ensure anonymity, no personal identifying information was collected from the participants.

### 2.2 Survey Design and Distribution

The survey was developed online using survey software (Qualtrics, Provo, Utah, USA). It was collaboratively designed by the research team comprising members with applied experience in professional male and/or female rugby. Specifically, lead author LW has provided nutrition support to an international female rugby union for the last 4 years, while NC and BJ have worked in professional male and/or female rugby for 3 and > 10 years, respectively. Additionally, SB contributes with > 15 years of research experience across a range of sports and governing bodies.

The survey underwent a pilot test by six Sport and Exercise Nutrition Register (SENr) accredited nutrition research‐practitioners with at least 3 years of experience within the sports industry. The pilot panel were asked to provide feedback on (a) the usability and technical functionality of the electronic questionnaire, (b) the survey structure and wording, (c) time to complete the survey, and (d) whether the questions related to the research purpose (Bolarinwa 2015). Based on the feedback, six questions and/or headings were altered. For example, a duplicate multiple‐choice option was amended, and section (e) was retitled from “application of nutrition theory to practice” to “application of behavioural theory to practice”. No questions were removed or added. To improve usability, ‘other’ was made an optional response on multiple choice questions, and a ‘submit’ button replaced a ‘forward‐arrow’ button, to ensure participants knew to submit responses.

The final version of the survey consisted of a minimum of 34 questions, and a maximum of 48 questions, because of the potential for 14 follow‐up and/or optional response questions. The survey was organised into eight sections: (a) participant information (i.e., job role, gender, code of rugby, accreditation status, academic qualifications, experience, and employment status); (b) nutritional knowledge and source of information (this section only applied to those who were not qualified nutritionists or dieticians, but were responsible for nutrition provision in their environment); (c) energy expenditure assessment (i.e., perceived importance, and methods used); (d) nature (i.e., practitioner roles and responsibilities) and content (i.e., topics of player support) of service provision (e.g., individual consultations, team education, menu development, travel nutrition, and supplement use), (e) behaviour change techniques (BCTs) (i.e., use, awareness, and perceived confidence in using them), (f) female specific practices (i.e., nutrition for the menstrual cycle) and (g) practitioner recommendations for future research. To ensure the data collected accurately reflected the sports nutrition services available to international rugby players, practitioners were instructed to answer questions based on their experiences within their current role in international rugby.

The questionnaire employed various question formats, such as multiple choice (*n* = 29), and bipolar Likert scale questions (*n* = 5). Some questions required participants to provide written responses (*n* = 14). The ordering of questions was not randomised per participant, as they followed a logical order.

There was a maximum of 10 questions per page, and the questionnaire was displayed over 10 pages (including consent page). Participants had the opportunity to review their answers prior to submission, using a ‘back‐button’. To ensure accessibility and convenience for participants, the survey was made available for completion on both desktop and mobile devices. Where necessary, the survey was translated for non‐English speaking unions.

### 2.3 Statistical Analysis

The statistical analysis was conducted using Microsoft Excel (version 2211, Excel 2019, Microsoft, Washington, USA). Descriptive statistics and frequency analysis were used to present responses from multiple‐choice and Likert scale questions, as the data in these cases consisted of either nominal or ordinal data. Likert responses have been combined (e.g., “*frequently”* and “*very frequently*”, alongside “rarely and “never”). Where appropriate, data are segregated by sex to facilitate comparative analysis. One participant worked with male and female players; their responses have been removed from gender comparisons to avoid error. Descriptive results are presented as mean ± standard deviation.

Content analysis was used to draw main themes from the data provided by open‐ended questions. Inductive content analysis initially identified the main themes through grouping and coding the data. Deductive content analysis was then performed to ensure all the raw data themes were represented (Elo et al. 2008; Heyward et al. 2020). All content analysis was performed independently by two researchers (L.W. and S.B.).

Only completed surveys were analysed. The survey had a completeness rate of 96 ± 3% (completeness rate: invited participants that answered every question). The survey took 36 ± 23 min to complete. This study adhered to the Checklist for Reporting Results of Internet E‐Surveys (CHERRIES) (Eysenbach 2004).

## 3 Results

### 3.1 Participant Information

Fifteen participants completed the survey, representing 16 international rugby unions. This included nine female and seven male teams (one participant worked with both a female and male union). Twelve of the unions recruited were ranked in the top 10 globally by World Rugby as of 10/01/2022. This included seven female and five male Top world 10 ranked teams. The sample included northern and southern hemisphere teams. Respondents included seven male and eight female participants, consisting of nutrition accredited practitioners (*n* = 12), S&C coaches (*n* = 2) and a team doctor (*n* = 1).

### 3.2 Employment Status and Service Provision

Participants were employed on a full‐time (*n* = 10/15), part‐time (*n* = 1/15), or contractual (*n* = 4/15) basis. Three international rugby unions did not employ a nutrition accredited practitioner, representing two female and one male union. Of the 12 accredited nutrition practitioners, 8 were employed full‐time. More nutritionists were employed on a full‐time basis when working for male (*n* = 4/5) versus female (*n* = 3/6) rugby unions. One nutrition practitioner was employed full‐time, across male and female teams of the same union.

**FIGURE 1:**
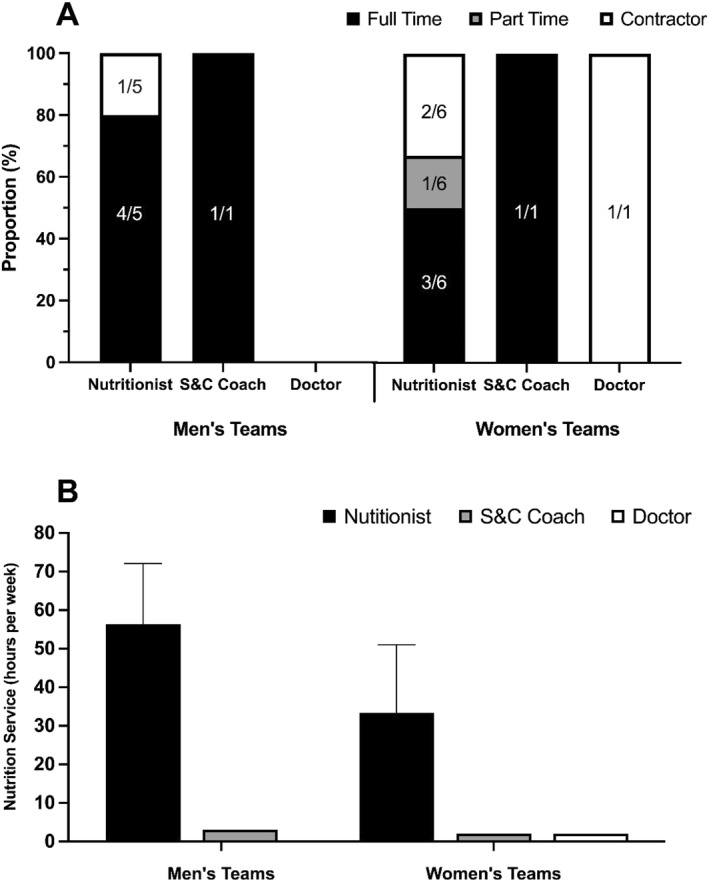
Comparison of (A) the employment status of the participants who deliver nutrition support, and (B) hours of nutrition service delivery per week to male and female international rugby unions. Numbers on bars represent the frequency of participants (*n*).

Nutrition practitioners provided more hours of nutrition service delivery (average hours per week) compared to sports science and medicine staff who were required to provide nutrition support (41.1 ± 22.5 vs 2.3 ± 0.6 h per week). Nutrition practitioners provided more hours of nutrition service delivery to male versus female unions (+23.1 h per week). An overview of participant employment status and hours of support provided is presented in Figure 1.

### 3.3 Education and Experience

**FIGURE 2:**
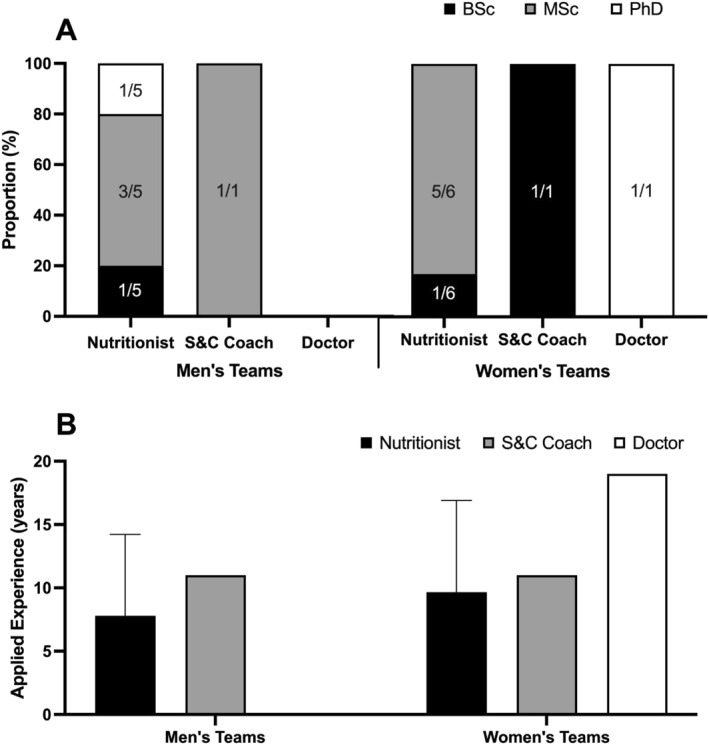
Comparison of (A) the education status, and (B) years of applied experience of the participants who deliver nutrition support to male and female international rugby unions. Numbers on bars represent the frequency of participants (*n*).

Participants had 11 ± 6 years of applied experience. The lowest and highest years of applied experience was 4 and 24, respectively. Of the 12 accredited nutrition practitioners, 9 had > 5 years of applied experience. All nutritionists reported having a nutrition related professional registration (e.g., SENr or equivalent). The lowest and highest level of education was a bachelor's degree (*n* = 3/15) and doctorate (*n* = 3/15), respectively. An overview of participant education and applied experience is presented in Figure 2.

### 3.4 Nutritional Knowledge

Among the three sport science and medicine (SSM) practitioners, one rated their nutritional knowledge as *‘good’*, one as *‘average’,* and one as *‘poor’*. SSM practitioners reported developing their nutrition knowledge from multiple sources, including ‘formal nutritional training as part of a wider degree programme’ (*n* = 2/3), and by ‘completing an online nutrition course’ (*n* = 2/3). No sport science and medicine practitioner had any form of nutrition accreditation. These participants reported using *‘scientific journals’* (*n* = 2/3), *‘internet articles’* (*n* = 1/3), *‘social media’* (*n* = 1/3), and *‘books’* (*n* = 1/3) as sources of nutrition information.

### 3.5 Nature of Service Provision

**FIGURE 3:**
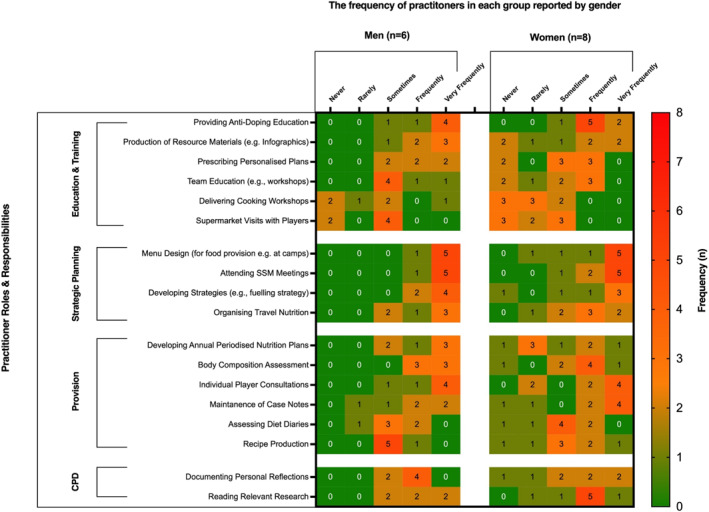
Nature of practitioner nutrition service provision to male and female international rugby unions. Values represent practitioner frequency (*n*).

Participants selected that they most frequently engaged with “*sport science and medicine meetings*” (*n* = 14/15), “*anti‐doping education*” (*n* = 13/15), “*menu design*” (*n* = 13/15) “*strategy development*” (*n* = 13/15), “*body composition assessment*” (*n* = 12/15) and “*individual player consultations*” (*n* = 12/15). Participants infrequently engaged with “*cooking workshops*” (*n* = 9/15) or “*supermarket visits*” (*n* = 8/15). An overview of the nature of participant service provision is displayed in Figure 3.

“*Other*” roles practitioners self‐reported included “*packing team nutrition supplies for training and competition*” (*n* = 2), “*attending training to provide recovery nutrition*” (*n* = 1), “*education of hotel staff for implementation of menus*” (*n* = 2), “*ordering supplements” (n = 1), “stock taking and allocation*” (*n* = 1), “*managing players hydration*” (*n* = 1), “*meeting with food and beverage brands around sponsorship*” (*n* = 2) and “*attending nutrition CPD events*” (*n* = 2).

Participants working with male unions more regularly engaged with “developing periodised plans” (male, *n* = 4/6 vs female, *n* = 3/8), “prescribing personalised plans” (male, *n* = 4/6 vs female, *n* = 3/8), “resource production” (male, *n* = 4/6 vs female, *n* = 3/8) and “body composition assessment” (male, *n* = 6/6 vs female, *n* = 5/8) compared to those working with female unions.

### 3.6 Content of Service Provision

Practitioners selected that they most frequently supported “*fuelling*” (*n* = 14/15), “*recovery*” (*n* = 14/15), “*injury rehabilitation*” (*n* = 14/15), “*meal and snack timing*” (*n* = 14/15), “*supplement use*” (*n* = 13/15), “in‐season nutrition” (*n* = 13/15), “*energy requirements*” (*n* = 12/15), “*hydration*” (*n* = 12/15), “*illness*” (*n* = 11/15), “*macronutrient requirements*” (*n* = 11/15) and “*body composition*” (*n* = 11/15). Participants less frequently supported “*disordered eating*” (*n* = 8/15).

**FIGURE 4:**
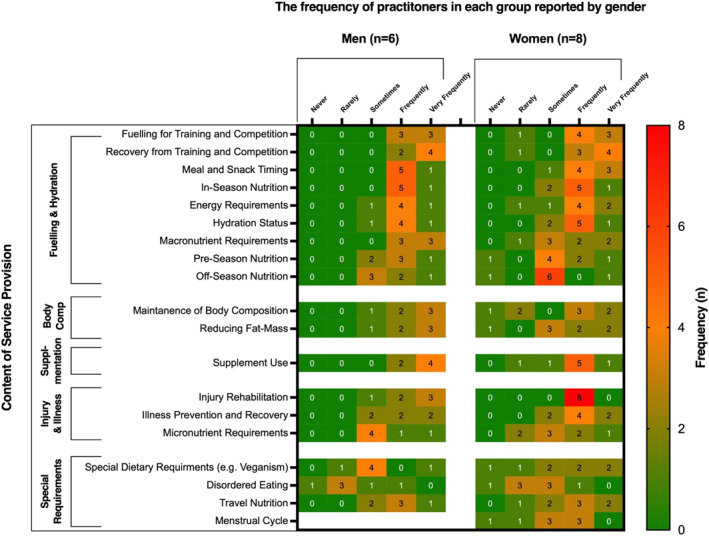
Content of nutrition service provision to male and female international rugby unions. Values represent practitioner frequency (*n*).

Participants working with female unions more frequently supported the “*menstrual cycle*” (male, *n* = 0/6 vs female, *n* = 3/8) and “*special dietary requirements*” (male, *n* = 1/6 vs female, *n* = 4/8) compared to those working with male unions. Participants working with male unions more frequently supported “*off‐season nutrition*” (male, *n* = 3/6 vs female, *n* = 1/8), “*macronutrient requirements*” (male, *n* = 6/6 vs female, *n* = 4/8), “*pre‐season nutrition*” (male, *n* = 4/6 vs female, *n* = 3/8) and “*body composition*” (male, *n* = 5/6 vs female, *n* = 5/8). An overview of the content of participant service provision is displayed in Figure 4.

### 3.7 Supplement Provision

Participants most frequently recommended “*caffeine*”, “*creatine*”, “*electrolytes*”, “*fish‐oils*” and “*whey protein*” (all: *n* = 13/15). Other commonly recommended supplements included “*collagen*” (*n* = 11/15), “*probiotics*” (*n* = 11/15), “*multi‐vitamins”*, “*sports drinks*”, “*sports gel*” and “*vitamin D*” (all: *n* = 10/15).

**FIGURE 5:**
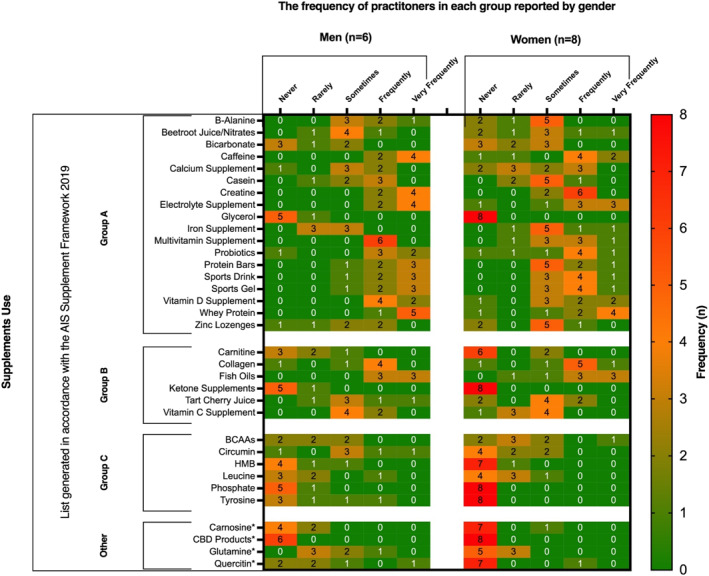
Frequency of supplement recommendations by participants working with male and female international rugby unions. Values represent practitioner frequency (*n*).

Participants working with female unions were more likely to recommended iron supplements (male, *n* = 0/6 vs female, *n* = 2/8) than those working with male unions. Participants working with male unions were more likely to recommend B‐alanine (male, *n* = 3/6 vs female, *n* = 0/8), casein (male, *n* = 3/6 vs female, *n* = 1/8), calcium (male, *n* = 2/6 vs female, *n* = 1/8), protein bars (male, *n* = 5/6 vs female, *n* = 3/8), multivitamins (male, *n* = 6/6 vs female, *n* = 4/8) and vitamin‐D (male, *n* = 6/6 vs female, *n* = 4/8). An overview of participant supplement provision is displayed in Figure 5.

### 3.8 Energy Expenditure Assessment

Of the 15 participants, quantifying the energy expenditure of rugby players was rated as *“extremely important”* (*n* = 2/15), *“very important”* (*n* = 10/15), and “moderately important” (*n* = 3/15). Most participants reported using predictive equations to quantify energy expenditure *“sometimes”* (47%, *n* = 7/15), whereas few reported using predictive equations *“very often”* (13%, *n* = 2/15), *“often”* (20%, *n* = 3/15), *“rarely”* (7%, *n* = 1/15), and *“never”* (13%, *n* = 2/15). Those that reported *“never”* had the role of team doctor and S&C coach. Predictive equations used by practitioners to estimate resting metabolic rate are presented in Table 1.

**TABLE 1:** Predictive equations used by participants to estimate player's resting energy expenditure.

Equation	Male unions (*n* = 6)^a^	Female unions (*n* = 8)^b^*	Male & female unions (*n* = 1)^c^
Schofield equation (1985)	0	3	0
Cunningham equation (1980)	4	3	1
Harris Benedict equation (1984)	4	5	1
Mifflin St Jeor equation (1990)	2	3	0
Henry Oxford equation (2005)	2	2	0
Other			
Ten Haff equation (2014)	0	1	0
GPS predictive energy expenditure	1	0	0

*Note:* Data are presented as frequency (*n*). ^a^5/6, ^b^4/8 and ^c^1/1 of the 15 participants reported using two or more predictive equations to quantify player energy requirements. *2/8 of participants reported ‘never’ using predictive equations to quantify players' energy requirements. NB. These equations are specifically used for the purpose of estimating a player resting metabolic rate.

Of the 15 participants, 4, 2, and 8 reported that predictive equations “underestimate”, *“neither over or underestimate”*, or *“both over and underestimate”* player energy expenditure, respectively. Fourteen participants accepted a small level of error (≤ ±200 kcal). Thirteen participants reported using multiple predictive equations, whereas 6 reported “taking an average of multiple equations”. Thirteen participants reported using ‘additional measures’ to increase their confidence in energy expenditure calculations. These included “body composition assessment” (*n* = 11/15), “body mass monitoring” (*n* = 6/15) and “dietary intake assessment” (*n* = 3/15).

Ten participants implemented a factorial approach to estimate athlete total energy expenditures (TEEs; resting metabolic rate multiplied by physical activity level). Participants reported that they were “*extremely confident*” (*n* = 1/15), “*very confident*” (*n* = 1/15) “*moderately confident*” (*n* = 6/15), “slightly confident” (*n* = 3/15) and “*not at all confident*” (*n* = 4/15) in TEEs estimated by a factorial approach. Low confidence was pertained to 3 main themes: “uncertainty over accuracy and reliability” (*n* = 6/15), “variability of training load, intensity and mode” (*n* = 5/15) and “accountability of individual characteristics, including muscle mass, training status, and contact involvement” (*n* = 6/15).

Of the 12 nutrition participants, 11 used energy expenditure calculations to inform service delivery. Participants reported using the data to “provide energy intake recommendations” (*n* = 8/12), “inform meal and snack suggestions” (*n* = 3/12), “provide periodised macronutrient recommendations” (*n* = 2/12), “produce team education resources” (e.g., infographics, *n* = 2/12), and “inform body composition manipulation” (*n* = 1/12). No sport science and medicine staff used energy expenditure data to inform service delivery.

### 3.9 Behaviour Change Techniques

Among the 15 participants, 14 were aware of and had used BCTs to influence athlete dietary behaviour(s). Participants reported that their BCT knowledge and application was developed from “*CPD events”* (e.g., conferences and training courses; *n* = 8/15), “*applied experience*” (*n* = 5/15), “*degree programmes*” (*n* = 4/15), “*reading literature*” (*n* = 4/15), and “*shared learnings*” (e.g., from other practitioners; *n* = 2/15).

Participants were “*moderately confident*” (*n* = 8/15) and “*very confident*” (*n* = 6/15) in using BCT's. Participants who were very confident in using BCTs attributed confidence to having a “*positive relationship with players”* (*n* = 2), “*seeing positive changes in players nutrition behaviou*rs” (*n* = 1), and “*length of career/experience*” (*n* = 5). Those who were less confident felt they needed “*more experience and training*” (*n* = 5).

The most common BCTs that participants reported using were “*goal setting (behaviour)*”, “*problem solving*”, “*re‐constructing the physical environment*”, “*salience of consequences*”, and “*self‐monitoring (outcome)*” (all: *n* = 12/15). The least used BCTs were “*feedback on a behaviour*” (*n* = 8/15), “*social* *support*” (*n* = 8/15), and “*demonstrating a behaviour*” (*n* = 7/15). Participant reported BCTs are presented in Table 2.

**TABLE 2:** Behaviour change techniques (BCTs) used by participants working with international male and female rugby union.

Behaviour change technique	Frequency [*n*]		
	Men (*n* = 6)	Women (*n* = 8)	Both (*n* = 1)
Demonstrating a behaviour (e.g., demonstrate how to cook a healthy meal)	2	4	1
Feedback on a behaviour (e.g., informing a player how many carbohydrates they consumed compared to their recommended requirements)	4	4	0
Goal setting (behaviour) (e.g., agreeing a target with an athlete to eat 5 fruit and vegetables per day)	4	7	1
Goal setting (outcome) (e.g., reduced illness incidence as an outcome of changed eating patterns)	3	7	0
Instruction on how to perform a behaviour (e.g., guide a player on how to select healthy meals when eating out at restaurants)	4	5	1
Problem solving (e.g., prompt player to identify barriers that prevent a behaviour and support them to identify a solution)	4	7	1
Prompts (e.g., send text to player to remind them to bring a fuelling snack to training or to increase carbohydrate intake on game day −1)	3	7	0
Provide information of health consequences (e.g., explain that not meeting carbohydrate requirements can compromise immune function and increase risk of illness)	3	7	0
Reconstructing the physical environment (e.g., changing food provision on match day to better suit athlete's nutritional requirements)	4	7	1
Salience of consequences (e.g., explain consequence of sub‐optimal nutritional intakes and provide evidence to support this e.g., days lost training or loss in muscle mass)	4	7	1
Self‐monitoring (behaviour) (e.g., asking a player to record a diet diary or take a picture of their recovery snack)	4	6	1
Self‐monitoring (outcome) (e.g., ask player to record their body mass weekly to increase weight loss behaviours)	4	7	1
Social support (e.g., ask family or partner to provide additional vegetables at meals to support player in meeting their nutritional requirements)	3	4	1
Using a credible source (e.g., present video recording of role model/high‐status professional athlete to emphasise the importance of good nutrition)	4	6	0
Other	0	0	0

### 3.10 Female Specific Practices

Of the 15 participants, 6 “*strongly agreed*” and 8 “agreed” that there is a lack of specific nutrition guidance for female rugby players. Of the nine participants serving women's unions, three, and four participants “*agreed*” and “*somewhat agreed*” that they rely on nutrition guidance for male rugby players in the absence of female evidence, respectively. Practitioners “*somewhat agreed*” (*n* = 9/15) and “*agreed*” (*n* = 3/15) that nutrition support is becoming more accessible within women's rugby. Among the 8 participants solely working with female rugby unions, 5 did not provide nutritional advise around the menstrual cycle.

### 3.11 Recommendations for Future Research

Participants' self‐reported recommendations for future research included 9 main themes: (a) the female player, (b) body composition, (c) energy requirements, (d) behaviour change, (e) macronutrient periodisation, (f) recovery, (g) supplementation, (h) collision and (i) injury. Practitioner suggestions for future research are presented in Table 3.

**TABLE 3:** Participants' suggestions for future nutrition research for international male and female rugby players.

Main category	Frequency [*n*]	Select raw data representing responses to this question
The female player	7	Female‐specific research is needed without question. Female health, for example, menstrual cycle, female specific nutritional needs.
Body composition	4	Case studies on players (positional) over the course of preseason and in‐season with physical targets (e.g., increase muscle mass, lower body comp etc.) Position body weight/composition normative values from underage to senior elite level. What a realistic amount of lean mass gain is over a period of time in female players.
Energy requirements	4	Risk of low energy availability in female rugby player. Energy availability in elite players
Behaviour change	3	Behaviour change, habit development, understanding and overcoming barriers, perception of nutrition roles among other performance staff. Behaviour change techniques for adherence to dietary guidelines.
Macronutrient requirements	3	Nutrition periodisation alongside training periodisation. Impact of contact on carbohydrate and protein requirements. Protein requirements in female athletes.
Recovery	2	Recovery—aids to recovery and pain management. Recovery strategies to mitigate soreness or speed up recovery.
Supplementation	2	The effect of probiotic supplementation on managing gut related concerns. Use of beetroot juice, beta alanine and caffeine in 7 s players.
Collision	2	Quantifying the collision better.
Injury	2	Injury specific nutritional recommendations for contusions, ligament and tendon injury and head injuries.

## 4 Discussion

Evaluating sport nutrition services in the context of international rugby is crucial to the advancement of service delivery and its practical application. Therefore, this study aimed to assess sports nutrition services available to international rugby unions, while exploring any disparities in nutritional support provided to male versus female players. Insights from 15 unions, featuring seven female and five male top 10 world ranked teams, revealed a reliance on both highly qualified staff and, in some instances, unaccredited personnel. Of note, male teams received more full‐time nutrition support than female teams, resulting in an average of 23 h more support per week. Practitioners predominantly focused on personalised fuelling, recovery, and hydration strategies, although there was a potential over emphasis placed on body composition manipulation, especially among male teams. The lack of female‐specific nutrition guidance was a concern among practitioners, many of whom resorted to male‐centric guidelines due to insufficient research for women. The study underscores the need for enhanced training in BCTs and calls for comprehensive research to improve nutrition services across all levels of international rugby, especially among female players.

### 4.1 Employment Status, Service Hours, Education and Experience

It is encouraging that most unions employed accredited nutrition practitioners (*n* = 12/15) with significant experience (over 5 years: *n* = 5/12, over 10 years: *n* = 4/12) and advanced qualifications (master's degrees: *n* = 8/12, doctorates: *n* = 2/12). Nevertheless, three unions depended on unaccredited staff for nutrition guidance, which may yield less than optimal advice, potentially detrimental to athlete health and performance (Beck et al. 2015; Burns et al. 2004; Bentley et al. 2019). Moreover, the relative lack of full‐time nutritionist roles (*n* = 8/12) in this survey might push athletes towards consulting less expert advice. Worryingly, a pronounced discrepancy was observed between male and female unions, with male unions employing more full‐time nutritionists, who provide 23 more hours of service per week. This gap suggests that female rugby players might receive less comprehensive nutrition services, pointing to a requirement for more investment in women's rugby to cater to their unique nutritional needs (Holtzman et al. 2021; Wohlgemuth et al. 2021). Ultimately, it is imperative that all rugby players, regardless of gender, have equitable access to qualified, accredited nutrition professionals.

### 4.2 Nature and Content of Service Provision

The findings confirm the critical role that nutrition practitioners have in international rugby, where they are key to both the design and implementation of dietary strategies. This involves a comprehensive range of services, including activities such as menu planning, co‐ordinating travel nutrition, and contributing to interdisciplinary meetings, with a predominant focus on fuelling, recovery, and hydration strategies, which align with athlete and research identified priorities (Solly et al. 2023; Jenner et al. 2021; Thomas, Erdman, and Burke 2016). Personalisation is key to their approach, highlighted by the frequency of individual consultations and tailored nutrition plans, which can also strengthen practitioner‐athlete relationships and adherence to dietary protocols (Solly et al. 2023; Jenner et al. 2021). Team activities such as nutrition workshops, cooking sessions and supermarket visits were completed less frequently, potentially due to the seniority of the investigated cohort, and a greater emphasis on provision of bespoke nutrition strategies over group‐based education. Interestingly, practitioners working with female unions produced fewer educational materials, such as infographics, potentially due to more pronounced time constraints. Importantly, the scope of sport nutritionists also covers specialised areas such as injury and illness management, emphasising the need for well‐qualified nutritional support to protect player health alongside performance.

Nutrition practitioners in international rugby play an important role in managing body composition, frequently conducting assessments and guiding athletes through changes. Optimally, such interventions should occur during off‐season and pre‐season phases to prevent any negative impact on performance from purposeful under‐fuelling (Meyer et al. 2013; Sundgot‐Borgen et al. 2013a, 2013b). Despite the typically brief duration of international competitions, these practices remain common, highlighting the need for better integration with club‐level practices. Interestingly, there is a lower incidence of body composition activities among female teams, possibly reflecting practitioners' heightened awareness of the risks associated with frequent body composition assessments in female athletes (or lack of appreciation of such risks within male cohorts) (McHaffie et al. 2022; Byrne et al. 2002; McMahon, Penney, and Dinan‐Thompson 2012; Ackerman et al. 2020). This could also reflect a difference in time allocation or the involvement of other sports science and medicine staff in these activities.

### 4.3 Supplementation

Nutrition practitioners in rugby commonly recommend evidence‐based supplements such as caffeine, creatine, electrolytes, fish oils and whey protein to support muscle recovery, reduce inflammation and enhance power and strength (Thomas, Erdman, and Burke 2016; Sport AIS). Creatine has also shown neuroprotective potential for concussive injuries (Dean et al. 2017; Roschel et al. 2021). Other supplements such as collagen, probiotics, multivitamins, sports drinks, gels, and vitamin D are frequently advised, whereas those with insufficient evidence or doping risks, such as HMB, phosphate, ketones, and CBD products, are avoided. These findings underscore the commitment of practitioners to evidence‐based supplement strategies which adhered to the Australian Institute of Sport's supplement framework (Category A and B) (Sport AIS). Moreover, practitioners exhibited sex‐specific supplementation practices, such as a higher prevalence of iron supplementation among those working with female players (Sims et al. 2023; Alaunyte, Stojceska, and Plunkett 2015).

### 4.4 Female Specific Practices

This study reveals significant challenges in female‐specific nutrition practices in rugby, with 14 out of 15 practitioners expressing concerns about the lack of female specific nutrition guidance tailored to rugby players. Furthermore, 7 out of 9 practitioners acknowledge that they rely on nutrition guidance intended for male players in the absence of dedicated female‐specific evidence. This reliance underscores the need for comprehensive research addressing the unique requirements and physiology of female athletes in rugby. Encouragingly, 12 out of 15 practitioners recognise that nutrition support is becoming more accessible within women's rugby. Interestingly, 5 out of 8 practitioners working with female players do not offer nutrition advice tailored to the menstrual cycle, possibly due to a gap in understanding regarding its impact on performance (McNulty et al. 2020) and the potential of cycle‐specific nutrition strategies to alleviate adverse effects in athletes (Sims et al. 2023). These findings further emphasise the urgency of developing high quality, evidence‐based, female‐specific nutrition guidelines, and further educating practitioners to support female rugby players in practice.

### 4.5 Energy Expenditure Assessment

Nutrition practitioners recognise the importance of assessing energy expenditure when optimising player health and performance (Thomas, Erdman, and Burke 2016), commonly using the Cunningham and Harris–Benedict equations for practicality. These equations, while cost‐effective, have their limitations; they only enable estimations of resting metabolic rate (a component of TEE) and may inaccurately estimate energy needs in both male and female players (Smith et al. 2018; Morehen et al. 2016; O'Neill et al. 2022). Predictive equations only enable estimations of resting metabolic rate Practitioners often incorporate additional metrics such as body mass and composition into their evaluations, accepting a minor error margin (≤ ±200 kcal) due to the complexities of direct resting metabolic rate measurement, and the precision that nutritional strategies can typically be delivered to within applied practice. Importantly, future research is now required to accurately establish the TEE of female rugby players (as already completed in men) (Costello et al. 2018a, 2019; Smith et al. 2018; Morehen et al. 2016), to support practitioners in coaching appropriate dietary energy and nutrient intakes in practice.

### 4.6 Behaviour Change Application

Changing athlete dietary behaviour(s) is an essential component of successful sports nutrition practice (Bentley, Mitchell, and Backhouse 2020; Costello et al. 2018b), with practitioners encouraged to utilise BCTs when coaching fundamental sport nutrition principles to athletes (Bentley et al. 2021). Among international rugby unions, 14 out of 15 practitioners have employed BCTs, focusing on “goal setting”, “problem solving”, “environment restructuring”, “salience of consequences” and “self‐monitoring”. These techniques have shown effectiveness in sports nutrition interventions (Bentley, Mitchell, and Backhouse 2020), including body composition changes in a professional male rugby league player (Costello et al. 2018b). However, practitioners' confidence in applying BCTs remains moderate (*n* = 8/15), with many seeking more training and applied experience; “I feel more experience of using BCT will increase my confidence” and “I probably need to upskill in this area for better outcomes”. Only 4 out of 15 practitioners acquired BCT knowledge through their degree programmes. To advance professional practice, educational pathways for sports nutritionists should emphasise BCT skills training (Bentley et al. 2021). Furthermore, professional governing bodies within sport nutrition should offer ongoing professional development to aid practitioners in effectively applying BCT theory in practice.

### 4.7 Limitations, Strengths and Areas for Future Research

This study, limited by a small but diverse international sample, sheds light on nutrition practices in elite rugby, with anonymity in data collection likely enhancing the validity of the self‐reported, expert‐informed responses. It draws on inputs from practitioners across various continents, including those from twelve top‐ten ranked rugby unions (five male and seven female), thus offering broad relevance and unique insights. Some respondents, not being registered nutritionists, could have affected the conclusions drawn. Moreover, the survey design and limited number of respondents precluded gender‐based statistical analysis. Many respondents offered additional information (on non‐forced response questions) as expert opinion and thus the accuracy of responses are not questioned. There is an absence of data on the frequency of complete recovery powder (i.e., carbohydrate and protein) use, and the frequency in which practitioners support players increasing levels of fat‐free mass. However, participants were provided the opportunity to add additional service provision details in open‐ended follow‐up questions. Information on catering provisions and efficacy of service provision was not collected, which would have been a worthwhile addition to the data set. Additionally, although data on hours‐of‐service provision were gathered, the weighing of provision between in‐camp and out‐of‐camp activities remains unclear. For the participant (*n* = 1) who worked across both male and female teams, the allocation of service hours to each team was not specified.

Future studies should investigate player and key stakeholder barriers and enablers to the implementation of high‐quality nutrition service delivery and precisely determine female rugby players' total energy requirements, supporting calls for more tailored research and guidelines for women in the sport. Such efforts would aid practitioners in designing effective interventions to enhance nutrition service delivery and support the unique needs of female (alongside male) players.

## 5 Practical Applications

The practical applications from this research extend to several key areas. Firstly, the findings underscore a requirement to employ qualified nutrition professionals within international rugby to oversee and deliver nutrition services. This ensures that players receive evidence‐based guidance tailored to their individual needs, fostering optimal performance and long‐term health. Secondly, gender‐specific nutritional guidelines and strategies should be implemented to ensure female player receive appropriate and equitable support. This may involve addressing unique physiological needs and dietary requirements specific to female rugby players. Thirdly, investment is required in practitioner education and training in BCTs to effectively support players in adopting and maintaining optimal nutritional practices. This includes empowering practitioners with the knowledge and skills necessary to address gender‐specific nutritional needs. Fourthly, service provision should move beyond a singular focus on body composition, particularly in male teams, towards a more holistic approach that encompasses player's overall health and wellbeing, alongside their performance. Finally, further high‐quality research aimed at advancing the practical delivery of nutrition services, particularly focusing on the unique requirements of female rugby players is required, as outlined in Table 3.

### 5.1 Conclusion

To conclude, this study offers a critical assessment of sports nutrition services in international rugby, identifying a notable discrepancy in the level of support between genders, with female athletes receiving less comprehensive services. Key issues include the reliance on male‐centric nutrition guidelines in female cohorts and a possibly unnecessary focus on body composition, especially in male teams. The findings advocate for enhanced practitioner education in BCTs and employment of qualified nutrition professionals to oversee nutrition services in practice. Finally, further high‐quality research is required to advance the practical delivery of nutrition services, especially for female rugby players.

## Author Contributions

L.W., B.J., S.H.B. and A.B. conceptualised the study. Data were collected and analysed by L.W. Data interpretation and manuscript preparation was undertaken by L.W., B.J., S.H.B., N.C. and A.B. All authors approved the final version of the manuscript.

## Ethics Statement

This study involved human participants and was approved by the Local Ethics Committee, Leeds Beckett University (84504).

## Consent

The authors have nothing to report.

## Conflicts of Interest

L.W. is responsible for nutrition service delivery to the National Women's Team at the Scottish Rugby Union (SRU). A.B. is head of athletic development at the SRU.

## Patient and Public Involvement

Patients and/or the public were not involved in the design, conduct or reporting of dissemination of this research.

## Supporting information

Supporting Information S1

## Data Availability

All data relevant to the study are included in the manuscript.
