# Supplementary material for: An Audit of Sport Nutrition Services Within Male and Female International Rugby Union: Implications for Research and Practice
Source: Eur J Sport Sci. 2025 Jan 30;25(2):e12260. doi: 10.1002/ejsc.12260 (PMC11781356; doi:10.1002/ejsc.12260)
Supplement: Supplementary file 1 — Supporting Information S1 [file EJSC-25-e12260-s001.docx]

**Supplementary Material 1: Copy of Survey**

**Understanding the Current Nutritional Services within Male and Female International Rugby Union**

**Section A – Participant Information**

Q1a. What is your sex? (A question about gender identity will follow)

Male

Female

Q1b. Is the gender you identify with the same as your sex registered at birth? (This question is voluntary)

Yes

No (please specify your gender identity) …………………………………………………………………………………………

Q2. Do you currently work with male of female rugby players? (Please select all that apply)

Male Players

Female Players

Q3. What is your hired role within the team?

Nutritionist/Dietician

Strength & Conditioning Coach

Team Doctor

Physiotherapist

Coach

Sport Scientist

Psychologist

Team Manager

Other (please specify) …………………………………………………………………………………………………………………….

Q4. Are you a nutrition/dietetics registrant of a professional body? (e.g., SENr, AfN, NSA, SDA, SNN) I.e., you are listed on a professional register which accredits your competency to apply evidence- based nutrition guidance to practice

Yes (please specify) …………………………………………………………………………………………………………………………

No

Q5. What is the highest degree or level of education you have completed?

No formal education

High School degree or equivalent

Bachelor’s degree e.g. (BA, BSc)

Master’s degree (e.g., MA, MSc, Med)

Doctorate (e.g., PhD)

Other (please specify) ……………………………………………………………………………………………………………………

Q6. Please indicate how many years of applied experience you have

Less than 1

1, 2, 3, 4 etc. (repeat until 30 years)

More than 30 years

Q7. What is your current employment status?

Employed full-time

Employed part-time

Self-employed/Contractor

Q8. Please specify the number of hours per week you assign to nutrition related roles (i.e., how many hours-of-service provision you provide).

…………………………………………………………………………………………………………………………………………………………….

**Section B – Participant Information (only displayed for participants who are NOT a nutritionist/dietician e.g., S&C Coach)**

Q1. Please indicate how you rate your knowledge of sports nutrition principles, using the options provided

Excellent

Good

Average

Poor

Very Poor

Q2. Please indicate where you source your information relating to sport nutrition (select all that apply)

Scientific Journals

Magazines

Internet Articles

Social Media (e.g., twitter, Instagram, Facebook)

Word of mouth

Common Sense

Video Platforms (e.g., YouTube)

Other (please specify) ………………………………………………………………………………………………………………………

Q3a. Have you ever received formal nutrition training?

Yes

No

Q3b. What type of nutrition training have you completed? (Only displayed if answered ‘yes’ to Q3a)

Part of a degree (e.g., module on an S&C degree)

Online course

CPD event

Other (please specify)………………………………………………………………………………………………………………………

**Section C – Energy Expenditure Assessment**

Q1. Quantifying the energy expenditure of rugby players is:

Extremely important

Very important

Moderately important

Slightly important

Not at all important

Q2. How often do you routinely use predictive equations to estimate energy requirements of players?

Very Often

Often

Sometimes

Rarely

Never

Q3. Please indicate what/which predictive equation(s) you use (select all that apply).

Schofield

Cunningham

Harris Benedict

Mifflin-St Jeor

Henry Oxford 2005

Not Applicable

Other (please specify) ……………………………………………………………………………………………………………………..

Q4. Please provide an explanation on your choice of predictive equation (optional)

…………………………………………………………………………………………………………………………………………………………….

Q5. Do you think predictive equations over or underestimate the energy requirements of your players?

Overestimate

Underestimate

Neither Over or Underestimate

Both Over and Underestimate

Unsure

Q6. Do you use any other measures or methods in triangulation with predictive equations? (e.g., weight assessment, body composition assessment)

Yes (please specify) …………………………………………………………………………………………………………………………

No

Q7. Does the use of additional measures increase the validity of data generated?

Yes

No

Unsure

Q8. Please provide any additional comments on your experience of using additional measures to improve validity (optional)

…………………………………………………………………………………………………………………………………………………………….

Q9. What level of error do you believe to be acceptable when using predictive equations to establish daily energy requirements?

less than +/- 100 kcal

+/- 100-200 kcal

+/- 200-300 kcal

+/- 300-400 kcal

+/- 400-500 kcal

more than +/- 500 kcal

Q10. What factors do you consider when determining the energy requirements of a player (select all that apply)?

Age

Body composition goals

Health condition

Illness

Injury

Menstrual cycle

Occupation

Phase of training cycle

Other (please specify) ……………………………………………………………………………………………………………………..

Q11. Are there any factors you believe predictive equations do not consider?

Yes (please specify) …………………………………………………………………………………………………………………………

No

Unsure

Q12. Do you apply a factor of physical activity level (PAL) to your predictive equations?

Yes

No

Unsure

Q13. How confident are you in quantifying a PAL for the players you work with?

Extremely confident

Very confident

Moderately confident

Slightly confident

Not at all confident

Q14. Please provide reasoning for your level of confidence?

…………………………………………………………………………………………………………………………………………………………….

Q15. Please describe how the data generated from predictive equations and PAL informs your practice?

…………………………………………………………………………………………………………………………………………………………….

Q16. Please describe how you share the data generated from predictive equations and PAL with players (optional)

…………………………………………………………………………………………………………………………………………………………….

Q17. Have you ever educated/trained a player to use predictive equations and PAL factors to estimate their own energy requirements

Yes

No

Q18. Please can you describe this process including the outcome (only displayed if answered ‘yes’ to Q17 – optional)

…………………………………………………………………………………………………………………………………………………………….

**Section D – Nature & Content of Nutrition Service Provision**

|  | **Strongly Disagree** | **Disagree** | **Neither agree nor disagree** | **Agree** | **Strongly Agree** |
| --- | --- | --- | --- | --- | --- |
| Nutrition support is essential to the programme and attainment of the players health goals |  |  |  |  |  |
| Nutrition support is essential to the programme and attainment of the players performance goals |  |  |  |  |  |
| Overall, players adhere to the nutrition guidance I provide |  |  |  |  |  |

Q1. Please indicate your responses to the following statements (use the following responses, strongly disagree, disagree, neither agree nor disagree, agree or strongly agree)

Q2. Please indicate the frequency you support the following nutritional aspects

|  | **Never** | **Rarely** | **Sometimes** | **Frequently** | **Very Frequently** |
| --- | --- | --- | --- | --- | --- |
| Disordered Eating |  |  |  |  |  |
| Energy Requirements |  |  |  |  |  |
| Fuelling for Training and Competition |  |  |  |  |  |
| Hydration Status |  |  |  |  |  |
| Illness Prevention and Recovery |  |  |  |  |  |
| Injury Rehabilitation |  |  |  |  |  |
| In-Season Nutrition |  |  |  |  |  |
| Macronutrient Requirements |  |  |  |  |  |
| Maintenance of Body Composition |  |  |  |  |  |
| Meal/Snack Timing |  |  |  |  |  |
| Menstrual Cycle* |  |  |  |  |  |
| Micronutrient Requirements |  |  |  |  |  |
| Off-Season Nutrition |  |  |  |  |  |
| Pre-Season Nutrition |  |  |  |  |  |
| Recovery from Training and Competition |  |  |  |  |  |
| Reductions in Fat Mass |  |  |  |  |  |
| Special Dietary Requirements (e.g., Veganism) |  |  |  |  |  |
| Supplement Use |  |  |  |  |  |
| Travel Nutrition |  |  |  |  |  |

*** menstrual cycle displayed only for those who reported working with female teams.**

Q3. Please specify any other nutritional aspects NOT listed above, that your frequently support (optional response).

…………………………………………………………………………………………………………………………………………………………

Q4. Which Supplements, if any, do you most frequently recommend and/or support a player’s decision to take (select all that apply) *please note, this list has been generated in accordance with the AIS sports supplement framework 2019.

|  | **Never** | **Rarely** | **Sometimes** | **Frequently** | **Very Frequently** |
| --- | --- | --- | --- | --- | --- |
| B-Alanine |  |  |  |  |  |
| BCAAs |  |  |  |  |  |
| Beetroot Juice/Nitrates |  |  |  |  |  |
| Bicarbonate |  |  |  |  |  |
| Caffeine |  |  |  |  |  |
| Calcium Supplement |  |  |  |  |  |
| Carnitine |  |  |  |  |  |
| Carnosine |  |  |  |  |  |
| Casein |  |  |  |  |  |
| CBD Products |  |  |  |  |  |
| Curcumin |  |  |  |  |  |
| Collagen |  |  |  |  |  |
| Creatine |  |  |  |  |  |
| Electrolyte Supplement |  |  |  |  |  |
| Fish Oils |  |  |  |  |  |
| Glutamine |  |  |  |  |  |
| Glycerol |  |  |  |  |  |
| HMB |  |  |  |  |  |
| Iron Supplement |  |  |  |  |  |
| Ketone Supplements |  |  |  |  |  |
| Leucine |  |  |  |  |  |
| Multivitamin Supplement |  |  |  |  |  |
| Phosphate |  |  |  |  |  |
| Probiotics |  |  |  |  |  |
| Protein Bars |  |  |  |  |  |
| Quercetin |  |  |  |  |  |
| Sports Bar |  |  |  |  |  |
| Sports Drink |  |  |  |  |  |
| Sports Gel |  |  |  |  |  |
| Tart Cherry Juice |  |  |  |  |  |
| Tyrosine |  |  |  |  |  |
| Vitamin C Supplement |  |  |  |  |  |
| Vitamin D Supplement |  |  |  |  |  |
| Whey Protein |  |  |  |  |  |
| Zinc Lozenges |  |  |  |  |  |

Q5. Please specify any other supplement NOT listed above, that you recommended to your players (optional response).

…………………………………………………………………………………………………………………………………………………………

Q6. Please indicate which practices you most frequently engage in

|  | **Never** | **Rarely** | **Sometimes** | **Frequently** | **Very Frequently** |
| --- | --- | --- | --- | --- | --- |
| Anti-doping education |  |  |  |  |  |
| Analysing diet diaries |  |  |  |  |  |
| Attending sport science and medicine team meetings |  |  |  |  |  |
| Body composition assessment |  |  |  |  |  |
| Delivering cooking workshops |  |  |  |  |  |
| Designing menus for food provision (e.g. at training camps) |  |  |  |  |  |
| Developing annual periodised nutrition plans |  |  |  |  |  |
| Developing protocols/strategies (e.g., fuelling strategy) |  |  |  |  |  |
| Documenting personal reflections and progress reviews |  |  |  |  |  |
| Improving shopping skills (e.g., supermarket visits) |  |  |  |  |  |
| Individual consultations/counselling with players |  |  |  |  |  |
| Maintenance of case notes of player interactions |  |  |  |  |  |
| Organising travel nutrition |  |  |  |  |  |
| Prescribing personalised meal plans |  |  |  |  |  |
| Production of resource materials (e.g., fact sheets) |  |  |  |  |  |
| Reading relevant literature to keep up to date on nutritional issues |  |  |  |  |  |
| Recipe production |  |  |  |  |  |
| Team based education (e.g., workshops) |  |  |  |  |  |

Q7. Please specify any other practices NOT listed above that you frequently engage in (optional response).

……………………………………………………………………………………………………………………………………………………………

**Section E – Behaviour Change Techniques**

Q1. Are you aware of any behaviour change techniques?

Yes

No

Not sure

Q2. Have you ever used a behaviour change technique to help a player change their dietary behaviour?

Yes

No

Not sure

Q3. Which of the following behaviour change techniques have you employed? (Select all that apply) [only displayed if answered ‘yes’ to Q2]

Demonstrating a behaviour (e.g., demonstrate how to cook a healthy meal)

Feedback on a behaviour (e.g., informing a player how many carbohydrates they consumed compared to their recommended requirements)

Goal setting (behaviour) (e.g., agreeing a target with an athlete to eat 5 fruit and vegetable per day)

Goal setting (outcome) (e.g., reduced illness incidence as an outcome of changed eating patterns)

Instruction on how to perform a behaviour (e.g., guide a player on how to select healthy meal when eating out at restaurants)

Problem solving (e.g., prompt player to identify barriers that prevent a behaviour and support them to identify a solution)

Prompts (e.g., send text to player to remind them to bring a fuelling snack to training or to increase carbohydrate intake on game day -1)

Provide information of health consequences (e.g., explain that not meeting carbohydrate requirements can compromise immune function and increase risk of illness)

Providing feedback on a behaviour (e.g., informing a player of how frequently they consumed protein compared to the recommended intake)

Reconstructing the physical environment (e.g., changing food provision on match day to better suite athletes’ nutritional requirements)

Salience of consequences (e.g., explain consequence of sub-optimal nutritional intakes and provide evidence to support this e.g., days lost training or loss in muscle mass)

Self-monitoring (behaviour) (e.g., asking a player to record a diet diary or take a picture of their recovery snack)

Self-monitoring (outcome) (e.g., ask player to record their body mass weekly to increase weight loss behaviours)

Social support (e.g., ask family or partner to provide additional vegetables at meals to support player in meeting their nutritional requirement)

Using a credible source (e.g., present video recording of role model/high-status professional athlete to emphasise the important of good nutrition)

Other (please specify) ……………………………………………………………………………………………………………………..

Q4. Please indicate where/how you developed your knowledge on behaviour change techniques (optional)

…………………………………………………………………………………………………………………………………………………………….

Q5. How confident are you in employing behaviour change techniques?

Extremely Confident

Very Confident

Moderately Confident

Slightly Confident

Not at all Confident

Q6. Please provide reasoning for your level of confidence? (optional)

…………………………………………………………………………………………………………………………………………………………….

**Section F – Female Specific Practices**

Q1. Do you support players in periodising their nutritional intakes during different phases of the menstrual cycle? (Only displayed to those who reported working with female players)

Yes

No

Unsure

Q2. Please give a description of what changes to dietary intakes you encourage during different phases of a player’s menstrual cycle (optional – only displayed if answered ‘yes’ to Q1).

----------------------------------------------------------------------------------------------------------------------------------------------------------------------------------------------------------------------------------------------------------------------------

Q3. Please indicate your responses to the following statements (use the following responses, strongly disagree, disagree, neither agree nor disagree, agree or strongly agree)

|  | **Strongly Disagree** | **Disagree** | **Neither agree nor disagree** | **Agree** | **Strongly Agree** |
| --- | --- | --- | --- | --- | --- |
| There is a lack of specific nutrition guidance for female rugby players |  |  |  |  |  |
| I often rely on nutrition guidance for male rugby players in the absence of female evidence |  |  |  |  |  |
| Sports nutrition support is becoming more accessible to women’s rugby |  |  |  |  |  |

**Section G – Recommendations for Future Research**

Q1. Please indicate what areas you believe future research should focus on, in order to develop the nutrition/dietary evidence base to support male and female rugby players health and performance

………………………………………………………………………………………………………………………………………………………………………………………………………………………………………………………………………………………………………………………….
